# New insights into the *Saccharomyces cerevisiae *fermentation switch: Dynamic transcriptional response to anaerobicity and glucose-excess

**DOI:** 10.1186/1471-2164-9-100

**Published:** 2008-02-27

**Authors:** Joost van den Brink, Pascale Daran-Lapujade, Jack T Pronk, Johannes H de Winde

**Affiliations:** 1 Kluyver Centre for Genomics of Industrial Fermentation and Department of Biotechnology, Delft University of Technology, Julianalaan 67, 2628 BC Delft, The Netherlands

## Abstract

**Background:**

The capacity of respiring cultures of *Saccharomyces cerevisiae *to immediately switch to fast alcoholic fermentation upon a transfer to anaerobic sugar-excess conditions is a key characteristic of *Saccharomyces cerevisiae *in many of its industrial applications. This transition was studied by exposing aerobic glucose-limited chemostat cultures grown at a low specific growth rate to two simultaneous perturbations: oxygen depletion and relief of glucose limitation.

**Results:**

The shift towards fully fermentative conditions caused a massive transcriptional reprogramming, where one third of all genes within the genome were transcribed differentially. The changes in transcript levels were mostly driven by relief from glucose-limitation. After an initial strong response to the addition of glucose, the expression profile of most transcriptionally regulated genes displayed a clear switch at 30 minutes. In this respect, a striking difference was observed between the transcript profiles of genes encoding ribosomal proteins and those encoding ribosomal biogenesis components. Not all regulated genes responded with this binary profile. A group of 87 genes showed a delayed and steady increase in expression that specifically responded to anaerobiosis.

**Conclusion:**

Our study demonstrated that, despite the complexity of this multiple-input perturbation, the transcriptional responses could be categorized and biologically interpreted. By comparing this study with public datasets representing dynamic and steady conditions, 14 up-regulated and 11 down-regulated genes were determined to be anaerobic specific. Therefore, these can be seen as true "signature" transcripts for anaerobicity under dynamic as well as under steady state conditions.

## Background

In the majority of industrial fermentation applications of bakers' yeast (*Saccharomyces cerevisiae*), a high initial and sustained capacity to ferment the available sugar is a highly important characteristic, especially when the biomass is introduced in an application environment with high sugar concentrations and/or absence of oxygen. Despite several attempts [1-3], quantitative data concerning the dynamics of the adaptation to such industrially relevant fermentative conditions have not been obtained. The majority of studies published to date on fermentative capacity under defined conditions rely on the use of batch or chemostat cultures [4-6]. The high specific growth rate in batch cultures does not reflect typical industrial conditions for aerobic cultivation of yeast biomass and has a drastic impact on fermentative capacity [7]. In chemostat cultures, which can be used in physiological studies to specifically investigate the effect of individual culture parameters, several physiological and transcriptional responses to the availability of oxygen and/or glucose have been identified [8-12]. However, in steady state chemostats dynamic responses to change in culture parameters can not be observed. A perturbation of one parameter in a chemostat cultivation results in a reproducible dynamic response from a defined constant culture [13]. By means of such experimental set-up, short and long term dynamics have been studied to pulses of low glucose concentrations [14,15].

The goal of the present study was to investigate the dynamic adaptation of *S. cerevisiae *to the industrially relevant transition from aerobic, sugar-limited and respiratory growth to fully fermentative (i.e., anaerobic glucose-excess) conditions and to dissect responses to the glucose up-shift and onset of anaerobicity. To this end, aerobic glucose-limited chemostat cultures grown at a moderate specific growth rate (0.10 h^-1^) were exposed to two simultaneous perturbations: a rapid depletion of oxygen and an increase of glucose concentration to a high value (40 g·l^-1^). Physiological analysis confirmed that the chemostat culture was fully respiratory before, and fully fermentative after the shift. Global dynamic responses to this combined perturbation were analyzed through genome-wide transcription analysis.

## Results and discussion

### Physiological characterization

**Figure 1:**
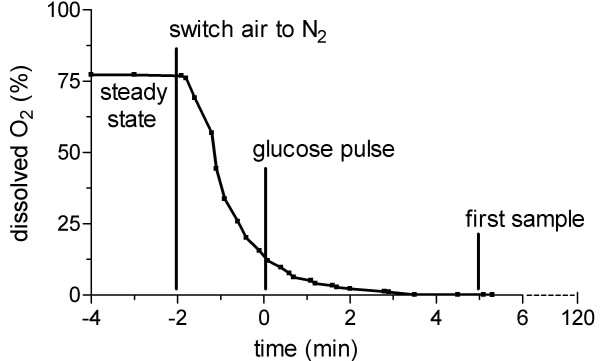
**Dissolved oxygen concentration during a shift to anaerobiosis**. Time zero corresponds with addition of glucose. The concentration is given in percentage of air saturation.

**Figure 2:**
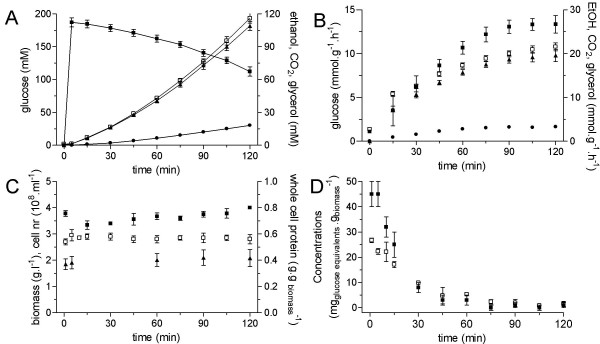
**Physiological responses of aerobic glucose-limited chemostat cultures to fully fermentative conditions**. Time zero represents the steady state value. **A **– Extracellular concentrations of glucose (black square), ethanol (black triangle), CO_2 _(open square) and glycerol (black dot). Each time point represents the average of at least six independent replicates. **B **– Specific rates of glucose consumption (black square) and ethanol (black triangle), CO_2 _(open square) and glycerol (black dot) production. **C **– Biomass dry weight (black square), whole cell protein (open square) concentrations and cell number (black triangle). **D **– Intracellular concentrations of trehalose (black square) and glycogen (open square). Each time point represents the average of at least two independent replicates.

To invoke rapid and full induction of fermentative capacity, respiratory, aerobic glucose-limited chemostat cultures (D = 0.1·h^-1^) were shifted to fully fermentative conditions by sudden depletion of oxygen and addition of glucose. The glucose was added two min after sparging the continuous culture with pure nitrogen, when the dissolved oxygen concentration had decreased from 75–80% to 10–15% of air saturation (Fig. 1). This raised the glucose concentration to 200 mM and ensured that the residual glucose concentration after 2 h of cultivation would still be above 100 mM, thus maintaining strong glucose catabolite repression throughout the experiment. (Fig. 2A) [16]. Indeed, the sudden shift to fermentative conditions resulted in fully fermentative metabolism within the first 5 min with CO_2_, ethanol and glycerol as the major metabolic products (Fig. 2). This metabolic shift coincided with an increasing specific glucose consumption rate, up to 12-fold, over 2 h following the perturbation (Fig. 2B). The specific ethanol production rate, which under these anaerobic glucose-excess conditions reflects the culture's fermentative capacity, steadily increased to 19.6 mmol ethanol·g^-1^·h^-1 ^(Fig. 2B). While the overall metabolic response was rapid and strong, the biomass concentration, the cell count and the cellular protein content did not change significantly throughout the experiment (Fig. 2C). During the two hours of the experiment, the growth rate did not exceed the starting growth rate of 0.1 h^-1^. The biomass therefore only contributed to 5% of the total carbon flux, while the main metabolic products (i.e. carbon dioxide, ethanol and glycerol) accounted for ca. 90% of the total carbon produced.

### Microarray data processing and general transcriptional response

To identify genome-wide transcriptional changes connected to the induced metabolic adaptation, micro-array analysis was performed on samples from two independent replicate steady-state chemostat cultures and on samples taken 5, 10, 30, 60 and 120 min after glucose addition. The coefficient of variation between replicates was below 20%, which is comparable with previous chemostat-based transcriptome analyses [10,11].

A first main concern was with normalization of these microarray data from non-steady-state culture samples. In previous transcriptome studies on steady-state chemostat cultures using Affymetrix microarrays, setting the average signal intensity of all probe-sets to a fixed value (also called global scaling) provided a good normalization method [10,11]. As this normalization method might not be appropriate for dynamic cultivation conditions, we considered transcript levels of a few so-called 'house-keeping' genes commonly used as loading standards for Northern analysis and quantitative RT-PCR. After global scaling, the expression of *ACT1*, *HHT2 *and *SHR3 *(encoding respectively, for actin, histone and endoplasmic reticulum packaging chaperone protein) remained constant throughout the experiment with a variation coefficient around or below 20%. The stable transcript levels of house-keeping gene expression obtained with a global scaling approach indicated that no major changes in the total mRNA pools occurred during the experiment, which would require another type of normalization.

**Figure 3:**
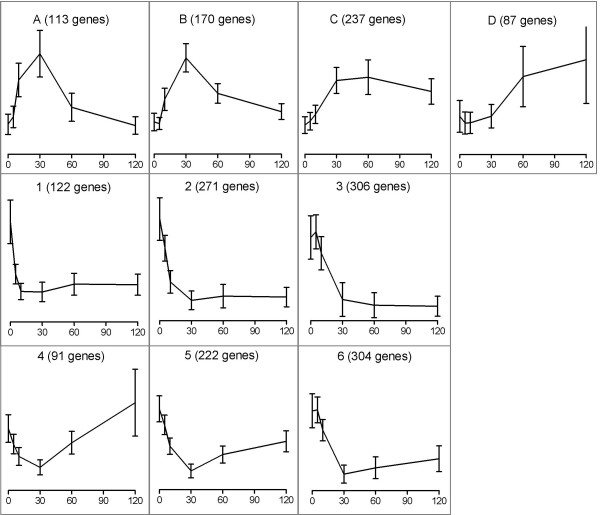
**Average time-dependent transcript profiles of clustered genes**. As described in Materials and Methods section, genes were clustered in 10 groups according to their initial and late transcriptional responses. Genes initially up-regulated were divided in 4 clusters called A, B, C and D, while down-regulated genes were allocated to clusters 1–6. Each line represents the average expression level of all genes in that cluster during the time course (0 (steady state), 5, 10, 30, 60 and 120 min). Absolute intensity values were mean normalized for each gene and for each time-point over all 13 arrays. Error bars indicate the standard deviation in normalized expression values of all genes in the cluster.

After global scaling, the significance of the changes in transcript levels during the dynamic experiment was estimated using the EDGE software (p-value threshold 0.005, [17], see Methods section for details). A set of 1923 genes was thus identified as being transcriptionally regulated in response to combined oxygen depletion and glucose addition (Additional file 1). This large group of genes was divided in several subgroups according to their expression profiles. 607 genes whose transcript levels increased after the perturbation were separated into four clusters according to their initial and later response (Clusters A-D; Fig. 3). 1316 genes with reduced transcripts responded rapidly to the perturbations (within 10 min) and were clustered according to their secondary response (Clusters 1–6; Fig. 3). All clusters were subsequently searched for overrepresentation of specific functional categories (as defined by MIPS [18]), and of promoter elements corresponding to specific transcriptional regulation networks (see Methods section). Upon a first inspection, some of clusters, despite subtle differences in their time-dependent transcript profiles, showed an overrepresentation of genes from the same functional categories. These were pooled to further improve the enrichment analysis. Thus cluster A and B, as well as 2 and 3, and also 4, 5 and 6 were pooled (Table 1 and 2), resulting in a final set of six different clusters.

**Table 1:** Overrepresentation of MIPS functional categories in genes that were differentially transcribed in response to fully fermentative conditions. Clusters A&B, 2 & 3 and 4, 5 & 6 had similar expression pattern and similar enrichments, and were therefore pooled before the overrepresentation analysis. Enrichment was estimated by hypergeometric distribution analysis (cut-offs around 10^-5^, see Materials and Methods); magnitude of the enrichment is indicated by the p-value.

**MIPS_cat**.			**A&B (283 genes)**		**C (237 genes)**		**D (87 genes)**	
nr.	description	nr of genes^a^	nr of genes^b^	p-value	nr of genes^b^	p-value	nr of genes^b^	p-value

01	**METABOLISM**	1531					38	3.8E-05
01.01	amino acid metabolism	243	28	2.6E-06	29	1.7E-08		
01.01.06	metabolism of the aspartate family	64			13	4.5E-07		
01.01.06.05.01	biosynthesis of methionine	14			5	1.0E-04		
01.03	nucleotide metabolism	230	23	6.3E-05	23	1.2E-05		
01.03.01	purine nucleotide metabolism	66			17	3.8E-09		
01.03.01.03	purine nucleotide anabolism	29			13	7.4E-12		
01.05.01	C-compound and carbohydrate utilization	510					18	1.4E-04
01.05.01.07.03	tetrahydrofolate-dependent C-1-transfer	14			6	5.8E-06		

11	**TRANSCRIPTION**	1036	126	9.4E-31				
11.02.01	rRNA synthesis	56	12	4.4E-06				
11.02.02	tRNA synthesis	39	9	3.8E-05				
11.04	RNA processing	394	89	9.4E-42				
11.04.01	rRNA processing	174	78	8.7E-62				
11.06	RNA modification	65	19	2.1E-11				
11.06.01	rRNA modification	17	9	1.0E-08				

12	**PROTEIN SYNTHESIS**	511	63	1.7E-14	103	7.6E-53		
12.01	ribosome biogenesis	343	49	8.5E-14	89	1.0E-54		
12.01.01	ribosomal proteins	277			88	1.8E-62		

14	**PROTEIN FATE (folding, modification, destination)**							
14.07.02	modification with sugar residues	70					8	4.1E-06
14.07.02.01	O-directed glycosylation	16					4	5.2E-05

16	**PROTEIN WITH BINDING FUNCTION**	1049	82	4.6E-08				
16.03	nucleic acid binding	346	47	2.0E-12				
16.03.03	RNA binding	194	31	2.8E-10				

20	**CELLULAR TRANSPORT, TRANSPORT FACILITATION**...	1038						
20.01.13	lipid transport	43					5	3.3E-05
**MIPS_cat**.			**1 (120 genes)**		**2 & 3 (577 genes)**		**4, 5 & 6 (617 genes)**	

nr.	description	nr of genes^a^	nr of genes^b^	p-value	nr of genes^b^	p-value	nr of genes^b^	p-value

01	**METABOLISM**	1531			193	4.3E-08	192	1.3E-05
01.05	C-compound and carbohydrate metabolism	510			89	2.6E-10	80	4.9E-06
01.05.01.01.01	sugar, glucoside, polyol and carboxylate catabolism	82					27	4.7E-09
01.06	lipid, fatty acid and isoprenoid metabolism	292					55	6.6E-07
01.06.01.07	isoprenoid metabolism	41					14	1.6E-05
01.06.01.07.11	tetracyclic and pentacyclic triterpenes biosynthesis	36					13	1.6E-05

02	**ENERGY**	360			112	5.1E-35	69	1.1E-08
02.10	tricarboxylic-acid pathway	31			11	4.7E-05	12	1.5E-05
02.11	electron transport & membrane-ass. energy conservation	54			30	4.2E-18		
02.13	Respiration	131			53	1.1E-22		
02.13.03	aerobic respiration	74			38	5.2E-21		
02.19	metabolism of energy reserves	53					16	2.4E-05
02.25	oxidation of fatty acids	9			6	3.5E-05		
02.45.15	energy generation (e.g. ATP synthase)	18					9	1.5E-05

11	**TRANSCRIPTION**							
11.02.03.04.01	transcriptional activator	42	7	1.2E-05				

14	**PROTEIN FATE (folding, modific., destination)**	1167					173	1.6E-08
14.07.11	protein processing (proteolytic)	92					24	4.1E-07
14.13	protein degradation	264					61	3.6E-11
14.13.01	cytoplasmic and nuclear protein degradation	194					48	4.2E-10
14.13.01.01	proteasomal degradation	134					33	2.8E-07

20	**CELLULAR TRANSPORT, TRANSPORT FAC**.	1028						
20.01.15	electron/hydrogen transport	76			24	2.3E-08		
20.09	transport routes	695					98	4.2E-05

32	**CELL RESCUE, DEFENSE AND VIRULENCE**	559			77	7.4E-05		
32.01	stress response	454			73	4.2E-07		
32.01.01	oxydative stress response	56			18	10E-06		
32.01.07	unfolded protein response (ER quality control)	74			16	7.6E-04		

42	**BIOGENESIS OF CELL. COMPONENTS**							
42.16	mitochondrion	170			38	9.0E-08	35	1.1E-05

a: amount of genes in the genome belonging to the specified functional category

b: amount of genes in the respective clusters or group of clusters belonging to the specified functional category

**Table 2:** Enrichment of transcription factors (TF) binding in clusters of genes that were differentially expressed in response to fully fermentative conditions. Clusters A & B, 2 & 3 and 4, 5 & 6 had similar expression pattern and similar enrichments, thereby these were analyzed together. Enrichment of TF binding according to the dataset of Harbison *et al*. [57] was given in p-value. Specific TF binding sites not present in the Harbison dataset (PAC, RRPE and Upc2p) were analyzed by using web-based software RSAT  and indicated in italics.

Transcription factor				A&B		C		D	
Name	cluster^a^	binding motif	nr of genes^b^	nr of genes^c^	enrichment^d^	nr of genes^c^	p-value	nr of genes^c^	enrichment^d^
Met32p	B	AAACTGTGG	22	6	2.94E-04				
Gcn4p	NC	TGAsTCA	192	22	3.54E-05	20	2.54E-05		
Bas1p	NC	TGACTC	36			14	1.19E-11		
Rap1p	NC	CAyCCrTrCA	157			49	2.79E-33		
Sfp1p	NC	AyCCrTACAy	51			25	5.25E-23		
Fhl1p	NC	TGTAyGGrTG	203			72	3.54E-54		
Gln3p	NC	GATAAGa	92					7	2.35E-04

*PAC*	*-*	*wGmGATGAGv [22]*	*376*	*98*	*5.7*				
*RRPE*	*-*	*TGAAAAwTTT [22]*	*535*	*110*	*4.2*				
*Upc2p*	*NC*	*TCGTwhAG [42]*	*667*					*16*	*1.7*

Transcription factor				1		2&3		4,5&6	
Name	cluster^a^	binding motif	nr of genes^b^	nr of genes^c^	p-value	nr of genes^c^	p-value	nr of genes^c^	enrichment^d^

Nrg1p	NC	GGaCCCT	128			25	1.59E-04		
Hap1p	NC	GGnnATAnCGs	73			25	1.75E-09		
Msn2p	5	mAGGGGsGG	65			20	5.57E-07		
Sut1p	A	GCsGsGnnsG	50			17	8.21E-07		
Skn7p	NC	GnCnnGsCs	156			37	2.56E-08		
Msn4p	5	mAGGGG	56			18	1.00E-06		
Hsf1p	NC	TTCynnnnnnTTC	133			31	5.38E-07		
Hap4p	3	GnCcAAtcA	54			16	1.31E-05		
Ash1p	NC	yTGACT	20			7	1.29E-03		
Sok2p	NC	TGCAGnnA	79			20	1.55E-05		
Ume6p	NC	TAGCCGCCsA	132			28	1.33E-05		
Rpn4p	3	GGTGGCAAA	93					21	1.60E-04
Mbp1p	NC	ACGCGT	165					44	1.75E-10
Swi6p	NC	CGCGAAAA	140					27	3.32E-04

a: presence of the transcription factor in one of the clusters; NC = transcription factor not significant changed over time

b: amount of genes in the genome belonging to the specified transcription factor

c: amount of genes in the respective clusters or group of clusters belonging to the specified transcription factor

d: enrichment represented by a p-value for the in-house analysis or a coverage coefficient (cluster coverage divided by genome coverage) for the analysis with RSAT tool

### Initial response

**Figure 4:**
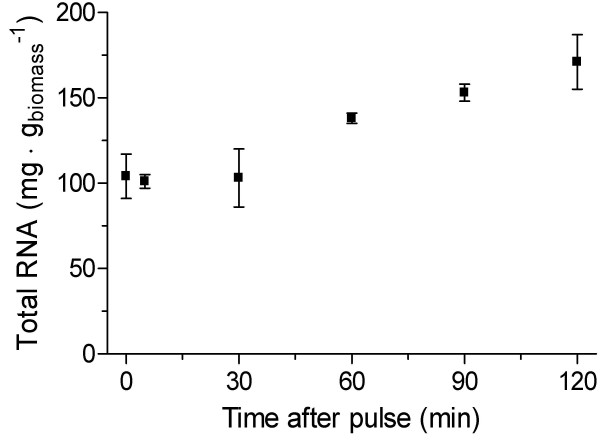
**Total RNA content of *S. cerevisiae *grown in aerobic glucose-limited chemostat before and after perturbation to fully fermentative conditions**. Time zero represents the steady state value. Each timepoint represents the average of at least two independent replicates.

Sudden relief from glucose limitation enables yeast cells to accelerate to a higher specific growth rate. Although faster growth was not observed in the 2 h after the relief of glucose limitation, over one third of the initially up-regulated genes were related to protein synthesis (Fig. 3; cluster A, B and C). This included a massive and fast up-regulation of genes within clusters A and B that encode components of the translational machinery, including 126 genes involved in rRNA synthesis, processing and modification and 49 genes involved in ribosomal biogenesis (Table 1). Taking into account that the total RNA pool mainly consists of rRNA [19], an up-regulation of rRNA synthesis was confirmed by an increase of the RNA content of the biomass after the relief from glucose limitation (Fig. 4).

Genes in cluster C displayed a sustained, slower increase of their transcript levels than those in clusters A and B. 37% of the genes in cluster C encoded ribosomal proteins. The delay between the expression of ribosomal biogenesis/rRNA genes and ribosomal protein genes is in line with previous observations indicating the existence of different regulatory mechanisms for these two groups of genes [20-22]. Accordingly, PAC and RRPE regulatory elements were enriched in the promoter regions of genes in clusters A & B, whereas Rap1p/Sfp1p and Fhl1p motifs were overrepresented in the promoter regions of cluster C genes (Table 2). In addition to the translational machinery, 57 genes involved in amino acid metabolism and 46 genes involved in nucleotide metabolism were up-regulated. This was consistent with the overrepresentation of Met32p [23], Gcn4p [24] and Bas1p [25] binding sites in the promoter regions of these genes, and indicated the need for synthesis of building blocks for transcription and translation.

Among the 1316 genes with reduced expression, one cluster comprising 122 genes showed rapid and strong repression (Cluster 1, Fig. 3). Although this cluster appeared relatively heterogeneous, one functional category was clearly enriched. It consists of seven transcription factor genes (*ACE2, PRP45, OAF1, GTS1, SWI5, MSN1 *and *STB1*) involved in various cellular functions, like fatty acid oxidation, stress response and cell cycle progression [26-31]. A large number of known targets of these transcription factors were also present in the down-regulated clusters (Additional file 2). Most of the remaining 1194 down-regulated genes were associated to metabolism and energy generation. In addition, a large number of genes involved in protein degradation (97 genes in total) were down-regulated, indicating a decreased requirement for proteolytic activity. Interestingly, 73 genes involved in stress response were down-regulated, including 18 related to oxidative stress response. This observation suggests that anaerobicity *per se *does not evoke an immediate stress for yeast.

### Secondary response

As expected, the initial response to fully fermentative conditions showed quite some overlap with published datasets for glucose pulses to aerobic cultures [14,32], including induction of the translational machinery and repression of the respiratory chain [33-35]. With this study, we aimed to go beyond the primary response to see how yeast adjusted to its altered growth environment.

**Figure 5:**
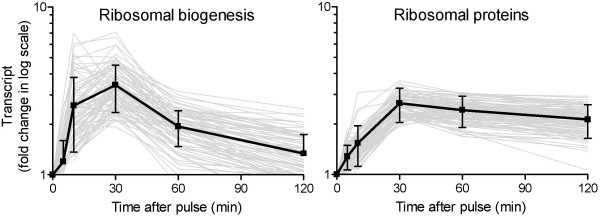
**The expression patterns of genes related to ribosomal proteins and ribosomal biogenesis**. The left panel represents the fold change compared to time point zero of all significant initially up-regulated genes belonging to the category of ribosomal biogenesis (MIPS 12.01), but did not belong to the category of ribosomal proteins (MIPS 12.01.01). The right panel represents the fold change compared to time point zero of all significant up-regulated genes belonging to the category of ribosomal proteins (MIPS 12.01.01). The profile of each gene is reported as a grey line while the average expression (plus standard deviation) is represented by the thick black line.

We did not identify genes whose transcript levels continuously increased or decreased in the 2 h following the perturbation. At 30 min after the shift, a pivotal point appeared to be reached at which the transcript profiles either indicated a reverse regulation mode (clusters A, B, 4, 5 and 6) or a stable mRNA level (clusters C, 2 and 3). In this respect, a striking difference was observed between the transcript profiles of genes encoding ribosomal proteins and those encoding ribosomal biogenesis components (Fig. 5). A steady transcript level after 30 min of ribosomal proteins was indicative for a constitutive requirement for translational building blocks to support faster growth. In contrast, transcriptional up-regulated genes involved in the synthesis, processing and modification of the translational machinery appeared only to be temporarily required for a rapid adaptation to the new environmental conditions. In addition to the ribosomal protein genes, genes involved in *de novo *purine biosynthesis, methionine metabolism, and tetrahydrofolate-dependent C_1 _metabolism were continuously transcribed at an elevated level after 30 min. All three functional categories have previously been correlated with each other, and with a response to the decrease in the adenine nucleotide pool [14].

Also the initially down-regulated genes with a turning point after 30 min could be divided in two groups: steady pattern after 30 min (clusters 2 & 3) or again up-regulated after 30 min (clusters 4, 5 & 6). During the course of the experiment, the glucose concentration remained high and hence, functional categories known to be repressed by glucose were enriched among the clusters in which the transcript level remained low after 30 min. Regulatory factors involved in regulation of the respiratory chain (*HAP2, HAP4 *and *HAP5*) were down-regulated together with their targets [36]. Stress-response genes also maintained low transcript level during the experiment. In contrast, transcripts of genes involved in lipid biosynthesis, reserve carbohydrate metabolism and protein degradation tended to increase again after 30 min. The large and coordinated transcriptional up-regulation of the translational machinery, specifically ribosomal proteins, complemented an opposite transcriptional regulation pattern of genes related to proteolytic activity. The down-regulation of target genes of the Mbp1/Swi6 complex, involved in G1 to S transition [37], correlated with a delay in cell cycle progression and correspondingly, a constant cell number over the two h monitored.

Many genes involved in the metabolism of storage carbohydrates (trehalose and glycogen) showed a decreased transcript level after the perturbation. To further investigate the observed changes in trehalose and glycogen metabolism, intracellular levels of trehalose and glycogen were measured. Both reserve carbohydrates were completely degraded within 30 min (Fig. 2D), consistent with a post-transcriptional activation of trehalose and glycogen phosphorylases [38,39]. Physiological interpretation of the trehalose and glycogen degradation however, is less straightforward, since trehalose and glycogen are known to be involved in flux regulation, stress response and cell cycle [39].

### Delayed responses related to anaerobiosis

Eighty-seven of the 1923 genes that showed a significantly altered transcript level after the combined glucose pulse and oxygen depletion only showed an increased transcript level after 30 min (cluster D). One of the few functional categories enriched within this group involved modification by glycosylation (*ALG7, GNT1, MNT4, OST5, PMT2, PMT4, PMT5, SEC53 *and *SWP1*). *PMT2*, *PMT4 *and *PMT5 *are specifically involved in O-linked mannosyl glycosylation, which is indispensable for cell wall integrity [40]. In addition, this 'delayed response' cluster contained five of the nine genes encoding anaerobically induced mannoproteins (*DAN1*, *DAN4, TIR1*, *TIR2 *and *TIR4*) [41]. Two other anaerobically induced mannoproteins (*DAN2 *and *DAN3*) were initially down-regulated, whereas transcript levels of the gene encoding the major cell wall mannoprotein (*TIP1*) did not significantly change at all.

A strongly anaerobiosis-related character of the genes in cluster D was not only suggested by the presence of the abovementioned genes involved in cell wall maintenance, but additionally by the presence of several genes involved in lipid transport (*AUS1, FAA4 *and *DNF2*), heme biosynthesis (*HEM13*; Rox1p repressed), sterol metabolism and regulation (*ARE1*, *HES1 *and *NCP1*), and cell wall biosynthesis (*EXG2*). Accordingly, a high number of genes contained AR1 elements in their promoter (Table 2), indicating a role of Upc2p [9,42]. The delayed up-regulation of these 'anaerobic genes' indicated that the response to anaerobiosis is slow compared to the fast response to the relief from glucose limitation (clusters A, B and C).

### Dissecting the response to anaerobiosis

The response to the anaerobic shift described in this study was compared with a dataset from a previously published study [20,21], in which the transcriptional response of batch cultures was monitored for several generations after a shift from aerobic to anaerobic conditions. Surprisingly, only 51 genes were overlapping with the significant up-regulated genes of our study. Half of these resided in our delayed response cluster D, which contains many anaerobiosis-related genes. The absence of a glucose pulse in the study of Lai *et al*. [20,21] explains the absence of genes encoding components of the translational machinery among the up-regulated genes in their dataset. Similarly, the large group of genes related to protein degradation found in the present study was not observed among the down-regulated genes identified by Lai *et al*. [20,21]. A strong overlap (464 genes) was found between the down-regulated genes identified in the two studies. Most of this overlap resided in the constitutively low expressed clusters 2 & 3 of our study (45% of the genes overlapped), which include many genes related to oxidative stress response. The majority of genes within the functional category Stress Response responded slower in the anaerobic shift study of Lai *et al*. [20,21] than in our study which included a step-up of the glucose concentration. Hence, we conclude that the observed regulation of stress response correlated with the relief from growth limitation rather than with a mere depletion of oxygen.

### Anaerobic "signature" transcripts

**Figure 6:**
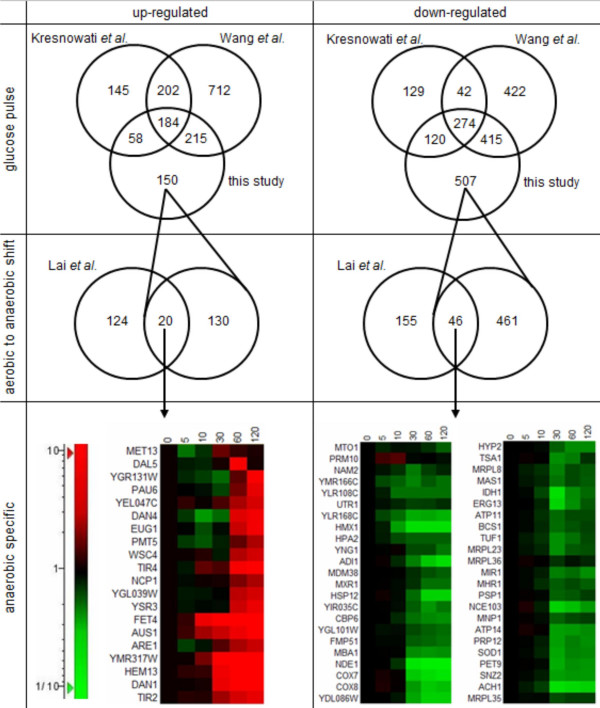
**Specific anaerobic genes determined by comparison between different dynamic studies with a glucose up-shift or shift from aerobic to anaerobic**. Up- or down-regulated genes of two different microarray studies with glucose pulses [14,32] were compared to the up-regulated genes within clusters A to D or to the down-regulated genes within clusters 1 to 6. The 150 up-regulated and 507 down-regulated genes not present in the previous glucose pulses [14,32] were compared with respectively 144 up-regulated and 201 down-regulated genes determined in the study of Lai *et al*. [20]. The heat maps represent the anaerobic specific genes given in fold change compared to time point zero.

In an attempt to identify robust 'signature transcripts' that show a consistent response to anaerobiosis, the set of significantly responding genes in this dynamic study was compared with several datasets from glucose pulses and aerobic-to-anaerobic shift experiments (Fig. 6)[14,20,32]. 457 genes of the 607 genes up-regulated in this study were previously identified in two other glucose-induced studies. Sixty-seven of the 150 non-overlapping genes resided within the delayed response of cluster D, indicating that more than 70% of the genes within cluster D were not responding to glucose. Twenty of these 150 genes are also up-regulated in the aerobic-to-anaerobic shift study of Lai *et al*. and can therefore been seen as specifically anaerobiosis-responsive (Fig. 6). These 20 anaerobic genes are involved in cell wall maintenance (DAN/TIR genes and related glycolysations), in membrane composition (*WSC4, DAL5 *and *FET4*) and metabolism (*HEM13, MET13, ARE1, AUS1 *and *NCP1*). Interestingly, 17 of those 20 genes resided in cluster D, and 12 genes contained the Upc2-binding promoter element (TCGTTTA), which earlier was associated with about 1/3 of anaerobic genes [9]. Transcription factor Upc2 has been reported to be strictly regulated by heme and sterol levels [43]. The delayed response of the anaerobic genes was likely due to the almost complete absence of growth during the experiment, thus sterol levels may not have been depleted rapidly through dilution over newly formed cells. A similar comparison with the down-regulated genes in our study resulted in 46 commonly responding genes (Fig. 6). The majority of the anaerobic down-regulated genes had functions related to mitochondrial function or oxidative stress response, of which 13 genes contained a Hap1 or Hap2/3/4/5 binding element [36,44]. Heme levels are likely to respond rapidly to the depletion of oxygen from the culture, consistent with the fast response of the anaerobically down-regulated genes.

The use of transcripts as a diagnostic tool for biotechnological applications has been proposed previously [8,11,45]. Based on steady-state cultivation experiments, a consistent response to anaerobiosis had been determined by analyzing aerobic and anaerobic chemostat cultures grown under different nutrient limitations (carbon-, nitrogen-, phosphorus-, and sulfur limitation), resulting in 65 anaerobically up-regulated and 90 down-regulated genes [11]. Surprisingly, only 14 up-regulated genes and 11 down-regulated genes were also found in both our dynamic study and the dynamic study of Lai *et al*.. Therefore, these can be seen as true "signature" transcripts for anaerobicity both within dynamic and steady state conditions (Table 3).

**Table 3:** "Signature" transcripts for anaerobicity within dynamic and steady conditions.

	Genes
Up-regulated	*ARE1, AUS1, DAN1, DAN4, EUG1, FET4, HEM13, PAU6, PMT5, TIR2, TIR4, YSR3*
Down-regulated	*ADI1, COX7, HMX1, MBA1, MSF1, NDE1, PRP12*, YDL086W, YGL101W, YIR035C, YLR108C

## Conclusion

We have studied the induction of yeast fermentative capacity by switching a fully respiratory culture to fully fermentative conditions. The aerobic glucose-limited chemostat culture with a low specific growth rate became, as seen in the physiology measures, fully fermentative for the entire experiment due to a rapid depletion of oxygen and addition of a high glucose concentration (40 g·l^-1^). The shift caused a massive transcriptional reprogramming, where one third of all genes within the genome were transcribed differentially. Our study demonstrates that, despite the complexity of this multiple-input perturbation, the transcriptional responses could be categorized and biologically interpreted. This required clustering of genes that shared discernable time-dependent responses to the perturbation, followed by a systematic analysis of overrepresented gene categories and upstream regulatory elements. This approach revealed that this reprogramming of the transcriptome was mostly driven by relief from the glucose-limitation, exemplified by preparation for faster growth (induction of ribosomes, nucleotide biosynthesis and amino acids biosynthesis) and glucose repression of various metabolic pathways. Contrary to previous observations [9,12], but as argued by Lai *et al *[20,21], the apparent relief from stress clearly indicates that anaerobicity *per se *does not evoke a stress in yeast.

A recent study by our group [14] studied transcriptional responses in the first five min after a glucose pulse to aerobic, glucose limited chemostat cultures. While that study revealed important and virtually instantaneous transcriptional events after imposition of a relief from glucose limitation, the present study shows that transcriptional reprogramming continues well beyond this 5 min period. Interestingly, most responses changed character after the 30 minutes point. This is clearly illustrated by the difference between the expression pattern of genes encoding ribosomal proteins versus genes encoding components for ribosomal biogenesis. Therefore, we have used this experimental set-up for further studying molecular details governing the observed differences in the regulatory mechanisms of the various groups of genes (manuscript in preparation).

One exception to the binary response mechanism observed around 30 minutes is presented by the anaerobic induction response, which appears only after the initial response to the glucose pulse. Most of the genes specifically induced by anaerobiosis are related to cell wall and plasma membrane remodeling. This is in contrast with Lai *et al*. where this response was only apparent after one generation [21]. The time span of anaerobic remodeling is therefore significantly shorter during a shift to complete fermentative metabolism on high glucose. By comparing this study with public datasets representing dynamic and steady conditions, the determined group of anaerobic "signature transcripts" will be better suited for use as a diagnostic tool in biotechnological applications.

Most of the transcriptional changes were due to sensitivity to the carbon supply. Still, the observed minor changes in transcripts for glycolytic enzymes cannot explain the 12-fold increase in flux through glycolysis under these conditions. Therefore we are presently studying the central carbon metabolism under such dynamic conditions by a multilevel approach, where transcripts, enzyme activities, metabolites and fluxes will be integrated. Hence, we will try to understand in more detail the regulatory mechanisms controlling fermentative capacity in yeast.

## Methods

### Strain and media

The *S. cerevisiae *strain used in this study was a prototrophic haploid reference strain CEN.PK113-7D (MATa) [46]. Stock cultures were grown at 30°C in shake flasks containing 100 ml of synthetic medium with 20 g of glucose per liter.

The synthetic medium contained per liter of demineralized water 5 g of (NH_4_)_2_SO_4_, 3 g of KH_2_PO_4_, 0.5 g of MgSO_4_·7H_2_O, 0.15 ml of silicon antifoam (BDH), and trace element concentrations according to Verduyn *et al*. [47]. After heat sterilization of the medium for 20 min at 120°C, a filter-sterilized vitamin solution [47] was added. The concentration of glucose in the reservoir medium was 7.5 g·l^-1^. This glucose was added to the synthetic medium after separate heat sterilization at 110°C.

### Chemostat cultivation

CEN.PK113-7D (MATa) was grown at 30°C in 2-l bioreactors (Applikon) with a working volume of 1.5 l via an electrical level sensor. Removal of effluent from the center of the culture ensured that biomass concentrations in the effluent line differed by less than 1% from those in the culture [48]. The dilution rate was set at 0.10 h^-1^. The pH was measured on-line and kept constant at 5.0 by the automatic addition of 2 M KOH using an Applikon ADI 1030 Biocontroller. A stirrer speed of 800 rpm and air flow of 0.75 liter·min^-1 ^were applied to keep the dissolved-oxygen concentration, as measured with an oxygen electrode, above 60% of air saturation in all chemostat cultivations performed. Steady-state samples were taken after ~10 volume changes to avoid strain adaptation due to long-term cultivation [49,50]. Biomass dry weight, metabolite, dissolved oxygen, and gas profiles were constant over at least three volume changes.

### Perturbation experiments

Anaerobic glucose-pulse experiments were started by sparging the medium reservoir of the fermentor of a steady-state glucose-limited aerobic chemostat culture (airflow of 0.5 liter·min^-1^) with pure nitrogen gas (Hoek-Loos, Schiedam, <5 ppm O_2_). Norprene™ tubing and butyl septa were used to minimize oxygen diffusion into the anaerobic cultures [51]. Two min after nitrogen sparging and just before adding the glucose, the medium-supply and effluent-removal pump was switched off. The 200 mM (60 g of glucose monohydrate in 60 ml water) glucose pulse was injected aseptically through a rubber septum. Samples were taken 5, 10, 30, 60 and 120 min following glucose addition.

### Analytical methods

The exhaust gas was cooled by a condenser connected to a cryostat set at 2°C and dried with a Permapure™ dryer (Inacom Instruments) before analysis of the O_2 _and CO_2 _concentrations with a Rosemount NGA 2000 analyzer. The gas flow rate was determined with an Ion Science Saga digital flow meter.

Acetate, ethanol, glycerol, and glucose concentrations in supernatants were determined by HPLC analysis with a Bio-Rad Aminex HPX-87H column at 60°C. The column was eluted with 5 mM sulfuric acid at a flow rate of 0.6 ml min^-1^. Acetate was detected by a Waters 2487 dual-wavelength absorbance detector at 214 nm. Glucose, ethanol and glycerol were detected by a Waters 2410 refractive index detector.

Culture dry weights were determined as described in [52] while whole cell protein determination was carried out as described in [53]. Cell numbers were counted by a Coulter counter (Multisizer II; Beckman Coulter) by using a 50 μm aperture.

### Trehalose and glycogen

Trehalose and glycogen concentration measurements were performed as described previously [54] in duplicate measurements on two independent replicate cultures. Glucose was determined using the UV-method based on Roche kit no. 0716251.

### Total RNA

Samples were collected during the pulse, washed three times with cold 5% trichloroacetic acid and the pellet is stored at -20°C. The samples were resuspended in 3% perchloric acid and heated at 90°C for 30 min. After centrifugation, the supernatant was mixed with 37% hydrochloric acid, containing 10 g l^-1 ^orcinol monohydrate (crystalline, Sigma-Aldrich, Germany) and 5 g l^-1 ^iron(III) chloride hexahydrate. The mixture was heated at 90°C for 20 min before measuring absorbance at 660 nm [55]. Absorbance values were related to a concentration (expressed as μg·ml^-1^) using a calibration curve of a standard yeast RNA solution (Sigma-Aldrich, Germany).

### Microarrays processing and analysis

Sampling of cells from chemostats, probe preparation, and hybridization to Affymetrix Genechip^® ^microarrays were performed as described previously [10]. The results for each time point after the perturbation (5, 10, 30, 60 and 120 min) were derived from two independently cultured replicates, while steady state data were derived from three independent chemostats. The complete dataset therefore comprised 13 arrays.

Acquisition and quantification of array images and data filtering were performed using Affymetrix GeneChip^® ^Operating Software version 1.2. Before comparison, all arrays were globally scaled to a target value of 150 using the average signal from all gene features. To eliminate insignificant variations, genes with expression values below 12 were set to 12 and genes for which maximum expression was 20 over the 13 arrays were discarded. From the 9335 transcript features on the YG-S98 arrays, a filter was applied to extract 6383 yeast open reading frames, as previously described [8]. To represent the variation in the measurements, the coefficient of variation was calculated as the mean deviation divided by the mean [8]. The array data used in this study can be retrieved at Genome Expression Omnibus [56] with series number GSE8187.

For additional statistical analyses, Microsoft Excel running the EDGE (version 1.1.208) add-in was used [17] for a time course differential expression analysis. To determine the genes called significantly changed according to EDGE a p-value of 0.005 was used. K-means clustering of the genes with significantly changed expression levels was subsequently performed using Genedata Expressionist^® ^Pro (version 3.1). The k-means algorithm used positive correlation as distance metric. The maximum number of iterations was set to 1000. Initially, the algorithm was run with k equal to 2, dividing the genes into an up- and a down-regulated cluster. Each cluster was then clustered again using k-means with k ranging from 2 to 10. The optimal k-value, i.e. 4 for the initially up-regulated and 6 for initially down-regulated genes, were based on the explained variance between clusters and the overrepresentation of functional categories (for detailed explanation please refer to Additional file 3).

Each cluster was consulted for enrichment in functional annotation and significant transcription factor (TF) binding (experimentally identified by Harbison *et al*. [57]) as described previously [58]. In addition, specific TF binding sites not present in the Harbison dataset were analyzed by using web-based Regulatory Sequence Analysis Tools [11,59].

## Authors' contributions

JvdB carried out the experimental work, interpreted the results and drafted the manuscript. PDL, JTP and JHdW contributed to conception and design. PDL interpreted the results and assisted in structuring the manuscript. JTP and JHdW assisted in structuring the manuscript. All authors read and approved the final manuscript.

## Supplementary Material

Additional file 1 **Table with all significant changed genes listed *per *cluster**. The Affymetrix ID, systematic name and the standard name were given for each gene. Click here for file

Additional file 2 **Figure with expression patterns of transcription factors Oaf1, Ace2, Swi5 and Gts1 (▲) and average expression patterns of their significant changed targets (□)**. Targets were all experimentally determined, as described at the proteome database of BIOBASE [60]. Expression values were given in fold change between each time point and time t = 0 (steady state). Click here for file

Additional file 3 **Quality measurements of the K-means clustering**. The k-values in a range from 2 to 10 were analyzed for its explained variance and the overrepresentation of functional categories. The quality of the individual clusters was measured by the same pairs proportion. Click here for file
