# Supplementary material for: New insights into the Saccharomyces cerevisiae fermentation switch: Dynamic transcriptional response to anaerobicity and glucose-excess
Source: BMC Genomics. 2008 Feb 27;9:100. doi: 10.1186/1471-2164-9-100 (PMC2292174; doi:10.1186/1471-2164-9-100)
Supplement: Additional file 3 — Quality measurements of the K-means clustering. The k-values in a range from 2 to 10 were analyzed for its explained variance and the overrepresentation of functional categories. The quality of the individual clusters was measured by the same pairs proportion. [file 1471-2164-9-100-S3.doc]

# Additional file 3

K-means clustering of the genes with significantly changed expression levels was performed using Genedata Expressionist® Pro (version 3.1). Initially, the algorithm was run with k equal to 2, dividing the genes into an up- and a down-regulated cluster. Each cluster was then clustered again using k-means with k ranging from 2 to 10. To determine the optimal k-value, we studied the explained variance for each k-value (Fig. 1).

The explained variance measures the fraction of the total variance of the data set that is due to the differences in the clusters. The explained variance increases when the number of clusters is increased. This is because with more clusters more parameters are used to explain the data, so that more of the variance in the data can be explained. Typically, when the number of samples equals the number of clusters, the explained variance is 1. A flattening of the increase of the explained variance indicates that the optimal number of clusters has been reached.

Deducted from this analysis, the k-value has to be at least 4 for the initially up-regulated and at least 6 for initially down-regulated genes (dashed line in Fig 1). Additionally, the k-values > 4 for the up-regulated and > 6 for the down-regulated clusters exceeds an explained variance of 0.8.

**Figure 1 –** Explained variance *per* k-value for the initially up-regulated and the initially down-regulated groups. Dashed line indicates the minimal k-value for further analysis. Calculations were performed by Genedata Expressionist® Pro (version 3.1).

To enable a biological interpretation of the clusters, clustering quality was assessed using functional enrichment. Therefore, we examined the overrepresentation of functional categories *per* k-value. The enrichment in functional annotation was performed as described in the Methods section.

Up-regulated cluster: the clustering with k = 4 resulted in the highest number of enriched categories (Table 2). The enrichment of the categories in the clusters (as indicated by their p-values) was furthermore not drastically different, i.e. higher or lower, for k>4. Therefore, we chose k = 4 for the up-regulated clustering, as this resulted in a good explained variance, the highest number of enriched categories and a good representation of MIPS category enrichment.

Down-regulated clusters: the down-regulated clusters contained around 28 different categories overrepresented irrespective of k (Table 3), moreover clustering with 4 < k < 10 gave similar enrichments of the categories. The main difference between the different k values was in the amount of clusters with no MIPS category overrepresented (Figure 2). We chose k = 6 for the down-regulated clustering, as this gave a low percentage of ‘empty’ clusters, good explained variance and a high number of enriched categories.

The 4 up-regulated clusters and the 6 down-regulated clusters were all tested for their quality by same pairs proportion, which measured how well a cluster was conserved in a second cluster. For each cluster it builds the set of pairs of observations that were in this cluster. Then it counts how many of these pairs were conserved in a second clustering. Ideally the number of conserved pairs was similar to the total number of pairs (proportion of 1). All the clusters had a proportion of minimally 0.55, indicating reasonable to good quality for each cluster.

**Figure 2 –** Percentage of clusters with no MIPS functional category overrepresented within the down-regulated group of genes.

**Table 1 –** Same pairs proportion of the 4 initially up-regulated clusters and the 6 initially down-regulated clusters. Calculations were performed by Genedata Expressionist® Pro (version 3.1).

| Cluster | Same Pairs Proportion | Cluster | Same Pairs Proportion |
| --- | --- | --- | --- |
| A | 0.564 | 1 | 0.800 |
| B | 0.599 | 2 | 0.699 |
| C | 0.921 | 3 | 0.665 |
| D | 0.699 | 4 | 0.557 |
|  |  | 5 | 0.571 |
|  |  | 6 | 0.600 |

**Table 2 –** Enrichment *per* k-value of MIPS functional categories for the up-regulated genes. The enrichment in functional annotation was performed as described in the Methods section. The enrichment was represented by the p-value. More than one p-value *per* category for one k indicated overrepresentation of the category in different clusters.

| **MIPS categories** | | **k=1** | **k=2** | **k=4** | **k=6** | **k=8** | **k=10** |
| --- | --- | --- | --- | --- | --- | --- | --- |
| nr. | description | p-value | p-value | p-value | p-value | p-value | p-value |
| 01 | METABOLISM | 3.3E-05 | 1.7E-06 | 3.8E-05 | 6.4E-05 |  |  |
| 01.01 | amino acid metabolism | 3.7E-13 | 5.6E-07 | 1.7E-08 |  | 2.9E-06 | 3.6E-05 |
|  |  |  | 7.1E-07 | 1.5E-06 |  | 3.2E-05 |  |
| 01.01.06 | metabolism of the aspartate family | 1.2E-09 | 1.6E-05 | 4.5E-07 |  |  |  |
|  |  |  |  |  |  |  |  |
| 01.03 | nucleotide metabolism | 2.4E-07 |  | 1.2E-05 |  |  | 1.1E-05 |
| 01.03.02 | purin nucleotide metabolism | 2.6E-09 | 2.6E-09 | 1.4E-10 | 3.8E-06 | 4.9E-09 | 6.2E-09 |
|  |  |  |  |  | 2.1E-05 |  |  |
| 01.03.01 | purine nucleotide metabolism | 8.7E-09 | 4.1E-10 | 7.4E-12 | 1.1E-07 | 2.5E-10 | 9.0E-07 |
|  |  |  |  |  |  |  | 2.0E-05 |
|  |  |  |  |  |  |  |  |
| 11 | TRANSCRIPTION | 1.8E-08 | 3.7E-29 | 2.7E-22 | 4.0E-16 | 3.4E-18 | 6.7E-14 |
|  |  |  |  | 1.2E-09 | 6.1E-13 | 7.9E-07 | 3.0E-09 |
|  |  |  |  |  |  |  | 8.9E-06 |
|  |  |  |  |  |  |  | 1.0E-05 |
| 11.02.01 | rRNA synthesis |  | 4.1E-06 | 1.4E-05 |  |  |  |
| 11.02.02 | tRNA synthesis |  | 3.6E-05 | 6.3E-05 | 2.8E-05 |  |  |
| 11.04 | RNA processing | 4.9E-21 | 4.3E-41 | 1.4E-23 | 7.3E-25 | 5.5E-26 | 1.6E-18 |
|  |  |  |  | 4.0E-18 | 8.1E-13 | 9.0E-09 | 3.1E-11 |
|  |  |  |  |  | 9.0E-06 | 9.0E-06 | 7.6E-11 |
| 11.04.01 | rRNA processing | 3.1E-43 | 4.7E-62 | 2.3E-33 | 2.2E-38 | 1.6E-39 | 1.0E-27 |
|  |  |  |  | 6.2E-26 | 3.7E-16 | 2.4E-11 | 1.1E-15 |
|  |  |  |  |  | 1.9E-07 | 1.9E-07 | 7.0E-14 |
|  |  |  |  |  |  |  | 1.4E-05 |
| 11.06 | RNA modification | 1.0E-08 | 1.8E-11 | 4.7E-13 | 3.4E-10 | 1.2E-05 | 3.5E-06 |
|  |  |  |  |  |  |  | 5.3E-06 |
| 11.06.01 | rRNA modification | 7.2E-06 | 9.8E-09 | 1.1E-10 | 6.3E-07 |  |  |
|  |  |  |  |  |  |  |  |
| 12 | PROTEIN SYNTHESIS | 1.4E-54 | 3.9E-40 | 7.6E-53 | 1.0E-56 | 8.9E-56 | 5.7E-42 |
|  |  |  | 4.2E-14 | 3.8E-08 | 1.6E-08 | 4.2E-09 | 3.4E-17 |
|  |  |  |  | 1.5E-07 | 1.7E-06 | 1.5E-07 | 1.5E-06 |
|  |  |  |  |  |  |  | 4.4E-05 |
| 12.01 | ribosome biogenesis | 2.6E-57 | 1.7E-41 | 1.0E-54 | 4.5E-60 | 1.0E-58 | 1.0E-47 |
|  |  |  | 1.5E-14 | 2.1E-08 | 1.2E-08 | 4.1E-09 | 5.0E-15 |
|  |  |  |  | 1.0E-06 | 1.5E-05 | 2.9E-05 | 7.3E-07 |
| 12.01.01 | ribosomal proteins | 2.3E-40 | 5.6E-48 | 1.8E-62 | 1.6E-67 | 6.3E-66 | 3.6E-52 |
|  |  |  |  |  |  |  | 2.6E-17 |
|  |  |  |  |  |  |  |  |
| 14 | PROTEIN FATE |  |  |  |  |  |  |
| 14.07.02 | modification with sugar residues |  |  | 4.1E-06 | 4.3E-07 | 3.4E-06 | 4.0E-06 |
| 14.07.02.01 | O-directed glycosylation, deglycosylation |  |  | 5.2E-05 | 1.6E-05 | 1.2E-05 |  |
|  |  |  |  |  |  |  |  |
| 16 | PROTEIN WITH BINDING FUNCTION |  | 1.6E-07 | 2.4E-08 | 4.1E-06 | 3.1E-05 | 1.1E-05 |
| 16.03 | nucleic acid binding | 4.7E-11 | 1.5E-12 | 1.5E-11 | 1.7E-08 | 2.0E-06 | 3.2E-08 |
| 16.03.03 | RNA binding | 2.0E-11 | 2.3E-10 | 3.3E-08 | 2.9E-07 | 3.4E-05 | 6.3E-06 |
|  |  |  |  |  |  |  |  |
| Nr. of different functional categories present | | 16 | 20 | 21 | 17 | 17 | 15 |

**Table 3 –** Enrichment *per* k-value of MIPS functional categories for the down-regulated genes. The enrichment in functional annotation was performed as described in the Methods section. The enrichment was represented by the p-value. More than one p-value *per* category for one k indicated overrepresentation of the category in different clusters.

| **MIPS catogeries** | | **k=1** | **k=2** | **k=4** | **k=6** | **k=8** | **k=10** |
| --- | --- | --- | --- | --- | --- | --- | --- |
| nr. | description | p-value | p-value | p-value | p-value | p-value | p-value |
| 01 | METABOLISM | 2.7E-13 | 7.1E-11 | 1.4E-05 |  | 1.2E-05 | 3.8E-05 |
|  |  |  |  | 5.8E-06 |  |  |  |
| 01.05 | C-compound and carbohydrate met. | 1.5E-15 | 7.6E-16 | 1.2E-09 | 3.2E-06 | 1.1E-05 | 3.6E-05 |
|  |  |  |  | 1.3E-06 | 4.2E-05 | 2.0E-05 | 3.9E-06 |
|  |  |  |  | 3.5E-05 |  |  |  |
| 01.05.01.01 | sugar, carboxylate metabolism | 2.0E-11 | 1.0E-10 | 1.9E-10 | 8.6E-09 | 1.7E-08 | 4.1E-08 |
| 01.05.01.01.01 | sugar, carboxylate catabolism | 1.4E-10 | 1.0E-09 | 1.0E-10 | 3.5E-08 | 1.2E-08 | 3.0E-08 |
|  |  |  |  |  |  |  |  |
| 01.06 | lipid, fatty acid, isoprenoid met. | 8.7E-08 | 2.5E-05 |  |  |  | 3.5E-05 |
|  |  |  |  |  |  |  |  |
| 02 | ENERGY | 3.6E-42 | 8.1E-50 | 2.1E-40 | 1.0E-29 | 1.3E-30 | 2.8E-05 |
|  |  |  |  | 5.5E-10 | 3.5E-07 | 7.7E-06 | 7.7E-11 |
|  |  |  |  |  | 3.2E-11 | 2.7E-10 | 1.9E-30 |
| 02.10 | tricarboxylic-acid pathway | 2.0E-10 | 1.5E-09 | 1.1E-05 | 9.4E-06 | 2.1E-05 | 8.1E-07 |
|  |  |  |  | 2.8E-05 |  |  |  |
| 02.11 | electron transport energy cons. | 1.0E-21 | 2.3E-27 | 2.7E-29 | 2.1E-23 | 3.5E-17 | 1.4E-08 |
|  |  |  |  |  | 9.5E-07 | 2.0E-09 | 1.1E-19 |
| 02.13 | respiration | 1.5E-23 | 1.9E-30 | 6.1E-28 | 2.2E-23 | 1.1E-22 | 6.7E-08 |
|  |  |  |  |  | 7.6E-07 | 8.0E-08 | 9.2E-22 |
| 02.13.03 | aerobic respiration | 2.6E-19 | 8.5E-24 | 2.9E-21 | 6.7E-20 | 6.1E-21 | 4.9E-19 |
|  |  |  |  |  |  |  |  |
| 02.16 | fermentation |  | 1.2E-05 | 1.7E-06 | 2.3E-07 | 7.3E-07 | 9.5E-06 |
| 02.19 | metabolism of energy reserves | 4.3E-08 | 1.9E-07 | 4.7E-05 | 2.4E-05 |  |  |
| 02.25 | oxidation of fatty acids |  | 3.0E-05 | 4.9E-07 |  | 3.6E-08 | 1.5E-08 |
|  |  |  |  |  |  |  |  |
| 02.45 | energy conversion and regeneration | 6.0E-07 | 1.1E-07 | 2.1E-08 |  | 7.7E-08 | 6.6E-09 |
| 02.45.15 | energy generation | 2.1E-08 | 8.5E-11 | 4.8E-10 | 3.7E-08 | 1.6E-12 | 1.1E-13 |
|  |  |  |  |  |  |  |  |
| 11 | TRANSCRIPTION |  |  |  |  |  |  |
| 11.02.03.04.01 | transcription activation |  |  | 1.6E-06 | 1.2E-05 | 7.5E-06 | 1.8E-05 |
|  |  |  |  |  |  |  |  |
| 14 | PROTEIN FATE |  |  | 1.5E-12 | 2.6E-05 | 2.6E-05 | 3.3E-07 |
|  |  |  |  |  | 1.6E-05 | 2.4E-07 |  |
| 14.07.11 | protein processing (proteolytic) | 3.3E-05 |  | 4.0E-08 | 5.5E-06 |  |  |
|  |  |  |  |  |  |  |  |
| 14.13 | protein/peptide degradation | 4.9E-10 | 1.0E-07 | 3.9E-14 | 3.2E-11 | 9.4E-06 | 4.8E-07 |
|  |  |  |  |  |  | 3.2E-06 |  |
| 14.13.01 | cytoplasmic and nuclear protein degr. | 3.1E-07 | 2.1E-05 | 7.8E-13 | 1.7E-09 | 2.1E-06 | 9.3E-08 |
| 14.13.01.01 | proteasomal degradation |  |  | 1.3E-09 | 5.8E-05 | 1.2E-05 | 3.9E-06 |
|  |  |  |  |  |  |  |  |
| 16 | PROTEIN WITH BINDING FUNCTION |  |  |  |  |  |  |
| 16.21 | complex cofactor/vitamine binding | 7.8E-06 |  | 2.2E-05 |  | 7.4E-06 | 1.9E-06 |
| 16.21.08 | Fe/S binding |  | 5.6E-05 |  | 2.5E-05 |  | 2.3E-05 |
|  |  |  |  |  |  |  |  |
| 20 | CEL. TRANSPORT, TRANSPORT FAC. | 3.1E-05 |  |  | 3.6E-05 | 4.4E-05 |  |
| 20.01 | transported compounds (substrates) |  |  | 3.3E-08 | 6.2E-07 | 5.9E-05 | 1.7E-05 |
| 20.01.11 | electron transport | 2.5E-11 | 1.7E-15 | 1.1E-17 | 3.4E-13 | 8.2E-09 | 1.1E-10 |
|  |  |  |  |  | 3.8E-07 | 4.7E-11 | 1.1E-09 |
| 20.09 | transport routes |  | 5.1E-05 |  | 7.2E-07 | 2.5E-07 | 1.2E-05 |
|  |  |  |  |  |  |  |  |
| 32 | CELL RESCUE, DEFENSE AND VIR. | 3.8E-05 | 4.5E-06 | 3.0E-06 | 3.9E-05 |  | 9.6E-06 |
| 32.01 | stress response | 3.4E-08 | 1.4E-08 | 3.9E-08 | 6.8E-07 | 4.8E-06 | 3.4E-07 |
| 32.01.01 | oxidative stress response | 4.8E-08 | 9.4E-10 | 2.2E-09 | 3.8E-09 | 1.6E-08 | 1.8E-07 |
| 32.07.07 | oxygen and radical detoxification | 6.3E-06 | 2.9E-08 | 1.4E-06 | 1.9E-05 | 4.1E-06 | 1.6E-05 |
|  |  |  |  |  |  |  |  |
| 42 | BIOGENESIS OF CEL. COMPONENTS |  |  |  |  |  |  |
| 42.16 | mitochondrion | 1.9E-13 | 1.3E-16 | 5.1E-08 | 9.4E-08 | 4.3E-07 | 7.3E-06 |
|  |  |  |  | 4.2E-08 |  | 1.2E-06 | 5.1E-05 |
|  |  |  |  |  |  |  | 1.0E-06 |
|  |  |  |  |  |  |  |  |
| Nr. of different functional categories present | | 25 | 27 | 29 | 27 | 27 | 29 |
